# Persistence of SARS-CoV-2 and its surrogate, bacteriophage Phi6, on surfaces and in water

**DOI:** 10.1128/aem.01219-23

**Published:** 2023-10-30

**Authors:** Ana K. Pitol, Samiksha Venkatesan, Michael Hoptroff, Grant L. Hughes

**Affiliations:** 1 Departments of Vector Biology and Tropical Disease Biology, Centre for Neglected Tropical Diseases, Liverpool School of Tropical Medicine, Liverpool, United Kingdom; 2 Unilever Research and Development, Port Sunlight, United Kingdom; Centers for Disease Control and Prevention, Atlanta, Georgia, USA

**Keywords:** SARS-CoV-2, bacteriophage Phi6, surrogate, survival, surface, fomite, water

## Abstract

The COVID-19 pandemic spurred research on the persistence of infectious SARS-CoV-2 and its surrogates, including bacteriophage Phi6, in environmental reservoirs. Despite the wide use of Phi6, side-by-side comparisons between Phi6 and SARS-CoV-2 are limited. Here, we quantified the persistence of SARS-CoV-2 and Phi6 on surfaces (PVC plastic and stainless steel), using an initial inoculum of 10^3^ plaque forming unit per surface, and evaluated the influence of four commonly used deposition solutions on viral persistence. In addition, we quantified the persistence of SARS-CoV-2 and Phi6 in water. Our findings revealed that Phi6 had a significantly longer half-life than SARS-CoV-2 in water and on surfaces. Phi6 persisted 34 hours in water compared with 13 hours for SARS-CoV-2. Viral persistence on surfaces was significantly influenced by the virus used and the deposition solution but not by the surface material. Phi6 remained infectious significantly longer than SARS-CoV-2 when the inoculation solution was culture media and saliva, leading to half-lives between 9 hours and 2 weeks for Phi6, compared to 0.5–2 hours for SARS-CoV-2. Using phosphate-buffered saline as a deposition solution led to half-lives shorter than 4 hours for both viruses on all surfaces. Our results indicate that bacteriophage Phi6 may lead to an overestimate of infectiousness for studies quantifying SARS-CoV-2 persistence on surfaces and water and highlight the importance of using appropriate deposition solutions when evaluating viral persistence on surfaces.

**IMPORTANCE:**

The COVID-19 pandemic spurred research on the persistence of SARS-CoV-2 and its surrogates. Here we highlight the importance of evaluating viral surrogates and experimental methodologies when studying pathogen survival in the environment.

## INTRODUCTION

SARS-CoV-2 is primarily transmitted through respiratory droplets and aerosols (1). Nevertheless, the RNA of SARS-CoV-2 has been extensively detected in environmental reservoirs, with concentrations as high as 10^5^ genome copies (gc) per swab on surfaces, and >10^5^ gc mL^−1^ in wastewater samples (2 – 7). The extensive contamination of environmental reservoirs with the SARS-CoV-2 RNA has been a source of concern throughout the COVID-19 pandemic (8). However, the concentration of SARS-CoV-2 RNA on surfaces is higher than that of infectious viral particles on a given sample (9, 10). Hence, using data on RNA contamination to estimate the risks associated with people interacting with contaminated environments could lead to overestimating the risks. Therefore, it is important to understand the persistence of infectious SARS-CoV-2 to estimate the magnitude of the risks associated with people interacting with contaminated environments.

Since the beginning of the pandemic, there have been a number of studies quantifying the persistence and inactivation of SARS-CoV-2 on surfaces and in liquids under different environmental conditions such as temperature and humidity (9 – 17). However, there is a lack of consistency in the experimental designs of these studies, which could lead to biased outcomes. For example, in studies of SARS-CoV-2 persistence on surfaces, a wide range of deposition solutions have been used to inoculate the virus, these range from culture media and bovine serum albumin (BSA)- containing media (16, 18) to bodily fluids such as human saliva and mucus (10, 19). The solutions used for virus inoculation have very different characteristics which can influence viral survival (20). In addition, working with SARS-CoV-2 requires handling the virus in high containment facilities, which presents many challenges and limits the type of experiments that can be performed. For example, it is not advisable to do experimental work using SARS-CoV-2 with human volunteers, such as testing SARS-CoV-2 persistence on human hands or quantifying the transfer of the virus between surfaces and hands.

Therefore, efforts have been made to use surrogates, instead of SARS-CoV-2, to understand the mechanisms of its survival, inactivation, and transfer (21 – 26). Bacteriophages are frequently used as surrogates of pathogenic viruses because they are safe, inexpensive, and do not require containment facilities (27 – 29). Bacteriophage Phi6 is one of the few bacteriophages which has a lipid envelope (30). Therefore, it has been used as a surrogate for enveloped viruses such as SARS and MERS coronaviruses, influenza virus, and Ebola (21 – 23, 29, 31 – 34). To this end, bacteriophage Phi6 has been used as a surrogate for SARS-CoV-2 in studies evaluating virus persistence on surfaces (23, 25, 26, 34), virus persistence in water and wastewater (35), virus inactivation (21, 22, 32), virus transfer from surfaces to hands (36), and virus recovery from fingertips (24). While these studies provide important information on viral persistence and transfer, there is a gap in our knowledge related to the adequacy of using bacteriophage Phi6 as a proxy for SARS-CoV-2. As such, there is an urgent need to evaluate the suitability of using bacteriophage Phi6 as a surrogate for SARS-CoV-2 in persistence studies.

Here we evaluated the persistence of SARS-CoV-2 and bacteriophage Phi6 in mineral water. In addition, we assessed the persistence of SARS-CoV-2 [10^3^ plaque forming unit (PFU)/coupon] and Phi6 (10^3^ PFU/coupon) on surfaces of two different non-porous materials: plastic (PVC) and metal (stainless steel). Since the deposition solution used to inoculate the virus on the surface could influence virus persistence, we evaluated four commonly used deposition solutions: phosphate-buffered saline (PBS), tryptic soy broth (TSB), cell culture media [Dulbecco’s minimal essential medium (DMEM)], and human saliva. Finally, we used the data to assess the suitability of using bacteriophage Phi6 as a surrogate for SARS-CoV-2 in studies of virus persistence in water and on surfaces.

## MATERIALS AND METHODS

### Bacteriophage Phi6 production and enumeration

Bacteriophage Phi6 (DSM 21518) and its host *Pseudomonas syringae* (DSM 21482) were obtained from the DSMZ (Deutsche Sammlung von Mikroorganismen und Zellkulturen). To culture the bacteriophage Phi6, we used a protocol adapted from Pitol et al. 2017 (37). Briefly, 100 mL of tryptic soy broth (TSB; Millipore) containing log-phase *P. syringae* was inoculated with 100 µL of a 10^8^ PFU mL^−1^ stock of bacteriophage Phi6 and incubated overnight. The following day, the media was centrifuged for 15 minutes at 5,000 rpm and the supernatant was filtered using a 0.45-µm filter unit. Aliquots of the supernatant with a final concentration of 1.5 × 10^11^ PFU mL^−1^ were stored at 4°C for subsequent assays.

To enumerate Phi6, we used the standard double agar layer assay (37). Briefly, a 4 mL aliquot of soft TSB agar (0.5% agar) was inoculated with 100 µL of an overnight culture of *P. syringae* at a concentration of 1.5 × 10^9^ CFU mL^−1^ and 100 µL of the sample containing an unknown concentration of Phi6. Samples were mixed and poured on hard agar (1.5% agar) TSB plates and incubated at 25°C overnight. Negative controls (100 µL of an overnight culture of *P. syringae* with no bacteriophage) were included in each experiment. All dilutions were quantified in duplicates, and all experiments were performed in triplicates.

### SARS-CoV-2 production and enumeration

SARS-CoV-2, Delta variant, passage 4 (SARS-CoV-2/human/GBR/Liv_273/2021, OK392641) (38) was amplified and quantified using Vero E6 cells (African green monkey kidney cells, Public Health England). Vero cells were maintained at 37°C and 5% CO_2_ in DMEM (Corning) supplemented with 10% fetal bovine serum (FBS; Sigma-Aldrich) and 0.05 mg mL^−1^ of gentamicin (Gibco). To amplify SARS-CoV-2, a flask T-150 of confluent Vero E6 in DMEM supplemented with 2% FBS was inoculated with 20 µL of a 10^6^ PFU mL^−1^ stock of SARS-CoV-2 Delta variant, passage 4, and incubated for 72 hours. Subsequently, the media was recovered and centrifuged for 15 minutes at 5,000 rpm to remove the remaining cells and cell debris. The recovered supernatant had a concentration of 10^7^ SARS-CoV-2 PFU mL^−1^. Because we were interested in comparing the persistence of SARS-CoV-2 using different deposition solutions, we wanted to minimize the carryover of growth media when diluting the stock solution of SARS-CoV-2 in the different deposition solutions. To do this, we proceeded to concentrate the stock of SARS-CoV-2 using the Amicon Ultra Centrifugal Filter (100 kDa; Merk Millipore Amicon) to a final concentration of ~10^8^ PFU mL^−1^. Before each experiment, the concentrated stock of SARS-CoV-2 (10^8^ PFU mL^−1^) was diluted 1:100 in each deposition solution, to a final concentration of 10^6^ PFU mL^−1^.

Standard plaque assay was performed to quantify infectious viruses as previously described (39). Briefly, samples were serially diluted and inoculated in a confluent monolayer of Vero E6 cells. One hour after infection, an agarose media overlay (2% agarose in DMEM supplemented with 2% FBS) was applied to the cell monolayer and incubated at 37°C and 5% CO_2_ for 72 hours. Subsequently, the cells were fixed with formalin 10% (VWR International), stained with crystal violet (Sigma-Aldrich) and plaques were counted (39). All experiments were conducted in a containment level 3 laboratory by personnel trained in the relevant code of practices and standard operating procedures.

### Persistence of SARS-CoV-2 and Phi6 in water

We selected bottled mineral water Volvic (Danone, France) for the persistence experiments, which is spring mineral water coming from a natural reserve in the Auvergne region of France that has been used elsewhere for similar experiments (40, 41). The mineral composition in the water is (mg/L): calcium 12; sulfates 9; magnesium 8; sodium 12; bicarbonates 74; potassium 6; silica 32; chlorides 15; nitrates 7.3; with a dry residue at 180°C of 130 mg and a liter pH of 7. The stock of 10^8^ PFU mL^−1^ of virus (SARS-CoV-2 or Phi6) was diluted to a final concentration of 10^6^ PFU mL^−1^ in Volvic water and aliquoted in samples of 50 µL in plastic cryotubes (Sarstedt), resulting in ~5 × 10^4^ PFU per sample. They were placed in an incubator with a controlled temperature (25°C) under dark conditions. The samples were recovered at time points of 0, 3, 6, 24, 30, 48, 72, 96 hours, and 1, 2, and 3 weeks. Subsequently, they were diluted in 200 µL of culture media (DMEM supplemented by 2% FBS) to a final volume of 250 µL, and stored at −80°C, before being quantified. Experiments were repeated three times.

### Surfaces and deposition solutions

To study the persistence of SARS-CoV-2 and Phi6 on surfaces, each virus was suspended in four different deposition solutions to a final concentration of ~10^6^ PFU mL^−1^. The solutions used were PBS (Gibco), TSB (Millipore), DMEM supplemented with 2% FBS, and human saliva. Saliva was collected at Unilever Research Port Sunlight as described elsewhere (38, 41). Briefly, healthy donors provided a stimulated daytime saliva sample for which they were given a piece of gum to chew (Wrigley’s Turbulence). Subjects were given a maximum of five sterile 30 mL containers in which they were asked to provide a 20–25 mL sample of saliva per container. Subjects were requested to wait 30 minutes after eating or drinking before providing a saliva sample. Saliva samples were stored overnight at −80°C prior sterilization using to gamma irradiation (Systagenix, UK, Cobolt 60 turntable, dose rate 1.2 kGy/h, minimum dose 32.1 kGy). After sterilization, saliva was stored at 4°C until used. All the deposition solutions used had a similar pH (TSB pH: 7.1–7.5, DMEM pH: 7–7.6, PBS pH: 7.4). The pH of saliva was not measured, but it has been reported that the saliva of healthy volunteers has a pH of 6.2–7.6 (42).

To study virus persistence on surfaces, we selected two non-porous materials: Plastic (PVC plastic vinyl) and metal (stainless steel). Circular coupons of stainless steel with an area of 3.14 cm^2^, and 1 cm^2^ square coupons of plastic were disinfected by soaking in 70% ethanol (VWR International) for 30 minutes. Subsequently, the coupons were thoroughly rinsed with deionized water before allowing them to dry inside a class II biological safety cabinet for 1 hour. They were placed in individual wells of a 24-well microtiter plate for the persistence experiments.

### Persistence of SARS-CoV-2 and Phi6 on surfaces

The plastic and steel coupons were inoculated by pipetting a 1 µL droplet of deposition solution (PBS, TSB, DMEM, and saliva) containing ~10^6^ PFU mL^−1^ of virus (SARS-CoV-2 or Phi6) in the center of the coupon, obtaining a final concentration of ~10^3^ PFU per coupon. The inoculated coupons were incubated at 25°C in a container partially opened to allow evaporation of the droplets, under dark conditions and 25–50% relative humidity. Coupons were sampled at different time points by pipetting up and down 15 times using 50 µL cell culture media (DMEM supplemented with 2% FBS) and samples were stored at −80°C before being quantified together at the end of the experiment. Positive controls, consisting of 50 µL DMEM inoculated with 1 µL of virus (SARS-CoV-2 or Phi6) suspended in each of the four viral matrices (PBS, TSB, DMEM, and saliva), were run alongside each treatment. Experiments were repeated three times. All experiments involving the use of SARS-CoV-2 were conducted in a containment level 3 laboratory facility at the Liverpool School of Tropical Medicine by personnel trained in the relevant code of practices and standard operating procedures.

### Data analysis

All data analysis was performed using R statistical software (version 4.1.0). Multiple regression analysis was used to assess the difference in the number of viruses in the water as a function of time and virus (Phi6, SARS-CoV-2). In the experiments that determined virus persistence on surfaces, multiple regression analysis was used to evaluate the survival of viruses as a function of virus, deposition solution (PBS, TSB, DMEM, and saliva), surface material (plastic, steel), and time.

Linear regression was used to calculate the first-order decay constant, k (h^−1^), as the slope of ln ( N / $N_{0}$ ) vs time in hours (equation (1)), where N is the number of viruses at time = *t*, and $N_{0}$ is the number of viruses at the beginning of the experiment (*t* = 0). Data with concentrations below the limit of detection (LOD) were excluded from the regression analysis. The decay constant, k , was used to estimate the half-life of the virus, $t_{50}$ (h^−1^), and the time required for 90% of the reduction in the number of viruses, $t_{90}$ (h^−1^). The $t_{50}$ and the $t_{90}$ were estimated using equations (2) and (3), respectively.

(Equation 1) $\ln\frac{N}{N_{0}}=-kt$

(Equation 2) $t_{50}=\frac{\ln2}{-k}$

(Equation 3) $t_{90}=$ $\frac{\ln10}{-k}$

## RESULTS AND DISCUSSION

### SARS-CoV-2 and Phi6 survival in water

**Fig 1:**
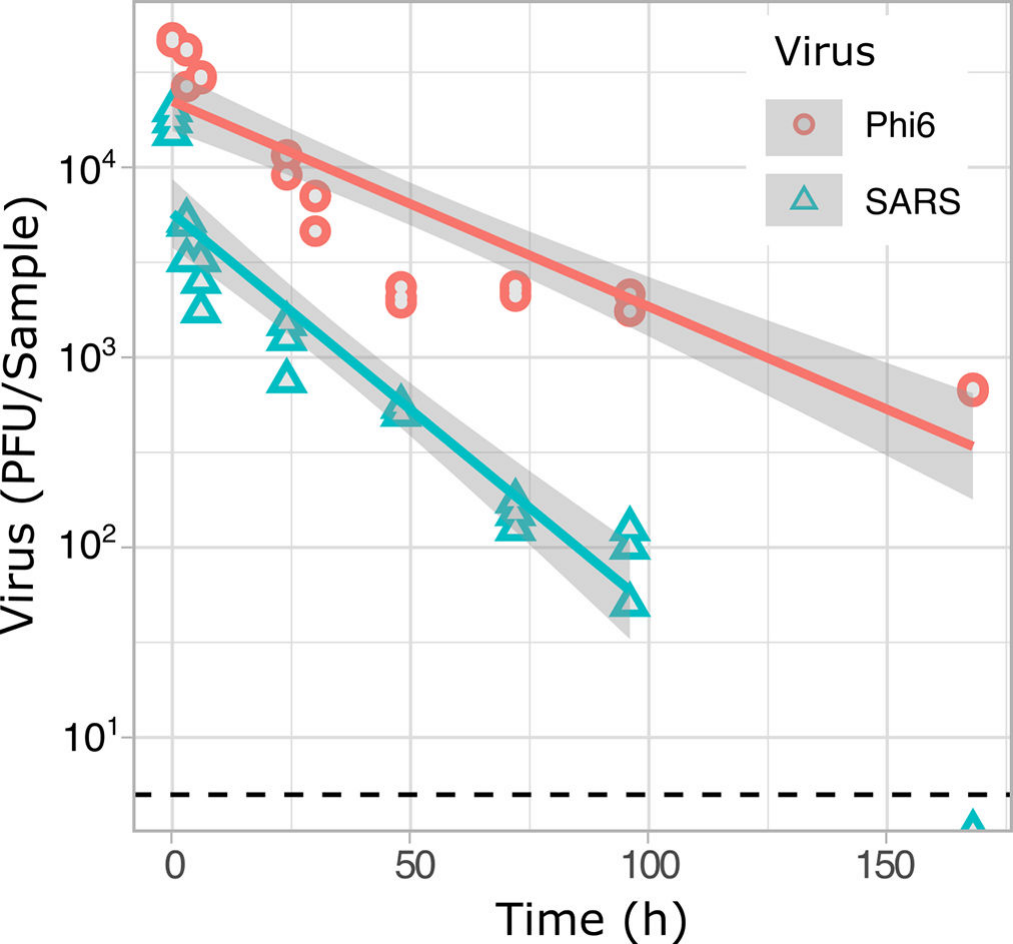
Persistence of SARS-CoV-2 and bacteriophage Phi6 in mineral water. The number of virus particles per sample are shown in red circles for Phi6 and blue triangles for SARS-CoV-2. The red and blue continuous lines show the fitted linear model for Phi6 and SARS-CoV-2, respectively, with gray areas showing the 95% CI for each regression line. The black dotted line shows the LOD for the assays, which was 5 PFU/sample. Each sample consisted of a 50 µL volume of water inoculated with the virus and incubated for specific times. Samples were placed in a dark incubator with a controlled temperature of 25°C and humidity ranging between 25% and 50%.

A multiple linear regression (MLR) was calculated to predict the concentration of viruses in the water as a function of time and virus type (*F* (2,47) =19.7, *P* < 0.001), *R2* = 0.43). The persistence of the viruses in water was significantly influenced by the virus used (MLR, *P* < 0.001). The half-life of bacteriophage Phi6 was 34 hours as compared with 13 hours for SARS-CoV-2 (Table 1; Fig. 1). Therefore, bacteriophage Phi6 significantly overestimated the persistence of SARS-CoV-2 in mineral water. The decay rates reported here for both viruses, Phi6 and SARS-CoV-2, fall within the range of data reported elsewhere (35, 43). The time taken to achieve a 90% reduction ( $t_{90}$ ) for SARS-CoV-2 in mineral water was 43 hours. Our findings were comparable to other studies using autoclaved or filtered river water and tap water at similar temperatures (20–25°C), $t_{90}$ = 48–79.2 hours (14, 15, 44). In addition, we obtained a $t_{90}$ of 112 hours for Phi6, which is consistent with previous research that shows a range of $t_{90}$ between 74 and 179 hours for autoclaved or filtered river water and tap water at similar temperatures (29, 35). The variation observed in virus survival between different studies can be explained by several factors that affect the chemical composition of the water, such as liquid pH (45, 46), salt concentration (45, 47), and the presence of polysaccharides (48) and proteins (26) in the solution, as well as environmental variables such as light exposure and temperature (26, 49). For example, studies of virus persistence on liquids have repetitively demonstrated that viruses are inactivated more quickly at higher temperatures (49). Therefore, a quantitative comparison between different studies is difficult, highlighting the importance of performing a side-by-side comparison between the viruses when evaluating the suitability of viral surrogates.

**TABLE 1:** Linear regression models for the persistence of SARS-CoV-2 and bacteriophage Phi6 in water

Virus	Liquid matrix	$t_{90}$ ^*a*^ (h)	$t_{50}$ ^*b*^ (h)	*K*, SE *k^c^* (h^−1^)
SARS-CoV-2	Mineral water	43.45	13.08	−0.053, 0.004
Phi6	Mineral water	112.42	33.84	−0.020, 0.002

^*a*^ Time required for 90% of the reduction in the number of viruses (hours).

^*b*^ Half-life or time required for 50% of the reduction in the number of viruses (hours).

^*c*^ First-order decay constant (hours^−1^).

### Persistence of SARS-CoV-2 and Phi6 on surfaces

**Fig 2:**
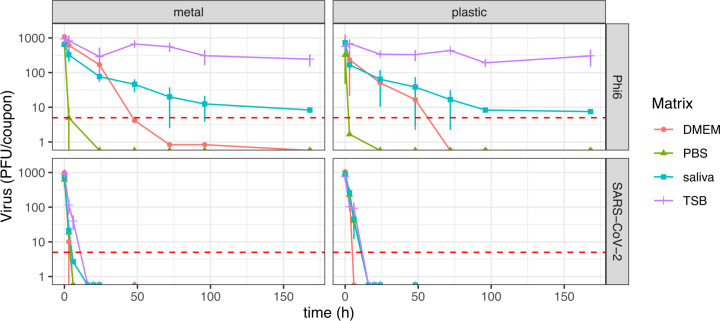
Virus survival on surfaces as a function of surface material (plastic vs metal), virus type (SARS-CoV-2 vs Phi6), and deposition solution (DMEM, PBS, TSB, and saliva). Data show the mean and the standard deviation of triplicates. The red dotted line shows the limit of detection of the assays, which is 5 PFU per coupon. Samples shown in the plot as below the LOD represent the average value of replicates with one or more data points > LOD. Samples were placed in a dark incubator with a controlled temperature of 25°C and humidity ranging between 25% and 50% for different time points.

MLR was used to predict the concentration of viruses as a function of time, virus type, deposition solution, and surface material (*F* (6, 376) =12.01, *P* < 0.001). Virus type, deposition solution, and time were significant predictors of virus concentration (MLR, virus type *P* < 0.001, deposition solution *P* < 0.001, time *P* < 0.001). Conversely, the influence of surface material (metal vs plastic) on virus persistence was not statistically significant (*P* = 0.57). The most marked observation to emerge from the data comparing the survival of SARS-CoV-2 with that of bacteriophage Phi6 on surfaces was that Phi6 survives significantly longer than SARS-CoV-2 (Fig. 2; Table 2). The half-life of Phi6 was 403 hours (~2 weeks) on plastic and 250 hours (~10 days) on metal when the deposition solution was TSB. Conversely, inoculating the surfaces with SARS-CoV-2 suspended in TSB resulted in half-lives of 1.3 and 1.9 hours for metal and plastic, respectively. Similar patterns of higher persistence of Phi6 as compared with SARS-CoV-2 were obtained when using saliva and DMEM as deposition solutions (Table 2). The half-life of Phi6 was 81 hours (~3 days) on plastic and 28 hours on metal when the deposition solution was saliva, as compared to 1.2 and 0.8 hours for SARS-CoV-2. Using DMEM as a deposition solution, the half-lives of Phi6 on plastic and metal were 12.3 and 9.3 hours, respectively, as compared with half-lives of 1.3 and 0.5 hours for SARS-CoV-2. Interestingly, when viruses were suspended in PBS, their half-lives were short, regardless of the virus used. Using PBS as a deposition solution led to Phi6 half-lives of 0.5 hours and 0.46 hours in plastic and metal, respectively, as compared to 1.3 and 0.6 hours for SARS-CoV-2.

**TABLE 2:** Summary of the mean linear regression model parameters for the persistence of SARS-CoV-2 and Phi6 on surfaces

Surface material	Deposition solution	SARS-CoV-2			Bacteriophage Phi6		
		*T*_90_ (h)	*T*_50_ (h)	*K*, SE *k* (h^−1^)	*T*_90_ (h)	*T*_50_ (h)	*K*, SE *k* (h^−1^)
Plastic	DMEM	4.24	1.28	−0.543, 0.125^*a*^	40.81	12.28	−0.056, 0.031
Plastic	TSB	6.35	1.91	−0.363, 0.080	1338.77	403.01	−0.002, 0.001
Plastic	PBS	4.27	1.29	−0.539, 0.042	1.66	0.50	−1.389, 0.000^*a*^
Plastic	Saliva	4.13	1.24	−0.557, 0.099	268.96	80.96	−0.009, 0.002
Metal	DMEM	1.63	0.49	−1.414, 0.086^*a*^	31.03	9.34	−0.074, 0.009
Metal	TSB	4.32	1.30	−0.533, 0.063	829.92	249.83	−0.003, 0.001
Metal	PBS	2.00	0.60	−1.150, 0.223^*a*^	1.51	0.46	−1.520, 0.086
Metal	Saliva	2.68	0.81	−0.859, 0.138	93.92	28.27	−0.025, 0.003

^*a*^ Only two time points were considered in the regression, as the virus was inactivated within 6 hours.

In addition to the higher persistence of bacteriophage Phi6 over SARS-CoV-2, our data indicate that the deposition solution had a significant influence on virus survival (Table 2; Fig. 2). This finding is consistent with previous research that suggests that the survival of viruses, including SARS-CoV-2 and Phi6, is highly influenced by the liquid matrix used to suspend the virus (20, 26, 45). Pastorino et al. showed that higher protein content in the deposition solution increases viral persistence on surfaces (20). In the present study, we used four commonly used deposition solutions: TSB (20 g of protein per liter, g L^−1^), human saliva (0.7–2.5 g L^−1^, based on data by Lin et al. ( 50)), DMEM supplemented with 2% FBS (~1.2 g L^−1^), and PBS (0 g L^−1^). Our data showed that bacteriophage Phi6 survives the longest in TSB, followed by saliva, DMEM, and lastly, PBS, which correlates with protein concentration in the deposition solution.

Another factor that could potentially influence the survival of viruses on surfaces is the initial concentration of virus inoculated on the surface. Bangiyev et al. studied the survival of Phi6 dried on plastic tubes using two deposition solutions: saline solution and culture media (Luria-Bertani (LB) growth medium) using four initial concentrations of Phi6 (26). They showed that a higher initial concentration of virus leads to increased half-lives when using saline as deposition solution, for example, when 10^4^ PFU of Phi6 in saline solution was dried on plastic the half-life was 57 minutes as compared with 5 minutes when the initial inoculum was 10^2^ PFU. By contrast, when using LB media, which has a high protein concentration (15 g of protein per liter), there was no observable difference in virus persistence at different initial virus concentrations (26). Similar to the results obtained when using Phi6 in LB medium, Paton et al. found no significant difference in the decay rate of SARS-CoV-2 on surfaces when inoculated at different starting concentrations (10^3^ vs 10^5^ PFU per surface) using culture media containing high protein content as deposition solution (51). This suggests a complex interplay between the initial concentration of the virus and the deposition solution on virus survival (51). In the present study, we inoculated surfaces with 1 µL of SARS-CoV-2 or Phi6 at a concentration of 10^6^ PFU/mL (10^3^ PFU/coupon) (50). The initial concentration of the virus used in this study is in line with those observed on the bodily fluids of patients with COVID-19 (52). Given the interplay between initial concentration and deposition solution, it is important to assess whether a high concentration of the virus can protect the virus from decay using biologically relevant matrices such as human saliva, which contain a significant amount of protein and show a protective effect on the virus, even higher than that observed using DMEM as deposition solution (Table 2). It is also worth noting that other biologically relevant matrices may have varying effects on virus persistence. For instance, respiratory mucus has been suggested to possess antiviral properties (53), and mucins, the primary proteins found in mucus, have been demonstrated to inhibit infection by human coronavirus OC43 (54). Therefore, it is advisable to conduct persistence experiments using a variety of relevant bodily fluids.

Our data on SARS-CoV-2 survival on surfaces are consistent with multiple research studies that showed that the half-life of SARS-CoV-2 at 20–27°C was between 1.5 and 9 hours in plastic surfaces (10, 13, 16, 55), and between 3.4 and 7.8 hours in stainless steel (9, 13, 16). Nevertheless, other studies have shown a longer survival rate for SARS-CoV-2 on surfaces at similar temperatures, with half-lives in the order of days rather than hours (18, 56). Differences between the studies include using different deposition solutions, initial inoculum concentrations, and drying times. Since experimental factors such as these mentioned above play a crucial role in the survival of viruses, it is imperative to carefully select these factors when evaluating the survival of emerging pathogens in the environment. This includes using an adequate deposition solution (e.g., bodily fluids such as saliva), and realistic virus inoculum concentration when designing experiments to evaluate virus survival in the environment.

We acknowledge our study has limitations, which are inherent when working with a category 3 pathogen such as SARS-CoV-2. For example, due to biosafety requirements, samples were placed in a container inside an incubator with no light or active air movement, which are factors that have been shown to influence virus survival (49, 57). Therefore, the decay of the viruses presented here may not adequately reflect the decay that viruses have in scenarios with sunlight exposure and air movement. In addition, experiments were performed at ambient humidity, which fluctuated between 25% and 50%. Temperature and humidity have been shown to influence the persistence of SARS-CoV-2 on the surfaces (13, 19). For example, one study showed that the half-life of SARS-CoV-2 on stainless steel ranges from 3 to 70 hours, depending on the environmental temperature and humidity (13). Therefore, adequate control of both temperature and humidity is advisable when evaluating the persistence of pathogens.

Despite the limitations of this study, our findings highlight the importance of evaluating the suitability of using viral surrogates by performing side-by-side comparisons with the pathogen of interest to control all variables that could potentially influence the outcome. Our results showed that, although it has been frequently used as a surrogate for coronaviruses, the use of bacteriophage Phi6 may lead to an overestimation of infectiousness for studies quantifying SARS-CoV-2 persistence. In addition, our findings reveal the need to use adequate deposition solutions when evaluating viral persistence on surfaces. Future research on the persistence of pathogens should place careful consideration on the methodology used, selecting a deposition solution, inoculation method, and environmental parameters that adequately mimic real-life scenarios.
